# Maximal Aerobic and Anaerobic Power Generation in Large Crocodiles *versus* Mammals: Implications for Dinosaur Gigantothermy

**DOI:** 10.1371/journal.pone.0069361

**Published:** 2013-07-05

**Authors:** Roger S. Seymour

**Affiliations:** School of Earth and Environmental Sciences, University of Adelaide, Adelaide, Australia; University of Pennsylvania, United States of America

## Abstract

Inertial homeothermy, the maintenance of a relatively constant body temperature that occurs simply because of large size, is often applied to large dinosaurs. Moreover, biophysical modelling and actual measurements show that large crocodiles can behaviourally achieve body temperatures above 30°C. Therefore it is possible that some dinosaurs could achieve high and stable body temperatures without the high energy cost of typical endotherms. However it is not known whether an ectothermic dinosaur could produce the equivalent amount of muscular power as an endothermic one. To address this question, this study analyses maximal power output from measured aerobic and anaerobic metabolism in burst exercising estuarine crocodiles, *Crocodylusporosus* , weighing up to 200 kg. These results are compared with similar data from endothermic mammals. A 1 kg crocodile at 30°C produces about 16 watts from aerobic and anaerobic energy sources during the first 10% of exhaustive activity, which is 57% of that expected for a similarly sized mammal. A 200 kg crocodile produces about 400 watts, or only 14% of that for a mammal. Phosphocreatine is a minor energy source, used only in the first seconds of exercise and of similar concentrations in reptiles and mammals. Ectothermic crocodiles lack not only the absolute power for exercise, but also the endurance, that are evident in endothermic mammals. Despite the ability to achieve high and fairly constant body temperatures, therefore, large, ectothermic, crocodile-like dinosaurs would have been competitively inferior to endothermic, mammal-like dinosaurs with high aerobic power. Endothermy in dinosaurs is likely to explain their dominance over mammals in terrestrial ecosystems throughout the Mesozoic.

## Introduction

The phylogeny of the archosaurs began in the Late Permian and diversified into two main lineages in the Middle Triassic, the Crurotarsi (crocodilians and their relatives) and the Ornithodira (dinosaurs, pterosaurs, birds and their relatives) [1]. Most palaeontologists now believe that birds evolved from dinosaurs in the Jurassic. So today we have crocodilians and birds as the surviving archosaurs, and these are sometimes considered to be an “extant phylogenetic bracket” that can be used to infer much about the status of dinosaurs. For example, Dodson [2] noted the obvious (“no-brainer”) conclusion that dinosaurs must have had 4-chambered hearts, because both crocodilians and birds do. Both birds and alligators have unidirectional flow in their lungs [3]. Crocodilians and birds also share many other anatomical features, including proteins, somatic muscles and bones, reproductive organs, sensory organs and behaviours such as maternal care and a vocal signalling repertoire [4], which set them apart from others in Clade Reptilia. It seems reasonable to accept that dinosaurs shared these features.

However, crocodilians and birds differ widely in their metabolic status: crocodilians are good ectotherms, behaviourally thermoregulate and have low metabolic rates, while birds are good endotherms, physiologically thermoregulate and have high metabolic rates. Extant phylogenetic bracketing is equivocal in this case, so there has been much debate about the metabolic status of dinosaurs. This paper cannot possibly include the literature relevant to the debate, but rather focuses on the implications associated with the proposal that ectothermic crocodilians represent a good model for dinosaurs, because large ones can behaviourally achieve high body temperatures and homeothermy at a low energy cost.

First it is necessary to define terms that this paper uses, because there is some confusion in the literature. *Endothermy* is the state in which metabolic rate is high enough and variable enough to permit physiological thermoregulation, resulting in body temperatures usually between about 32–40°C. *Ectothermy* is the state in which metabolic rate is low, so that thermoregulation (if at all) is largely behavioural manipulation of heat input from the environment, principally the sun. *Homeothermy* is the maintenance of a stable body temperature, at any level and without any essential connection with metabolic rate or endothermy. Animals can be homeotherms if they are capable of physiological thermoregulation, or are in a thermally stable environment, or are large enough to buffer environmental temperature changes (“inertial homeothermy” or “gigantothermy” as coined by Paladino et al. [5]). Gigantothermy is real, because it is based in physics. It has been predicted by mathematical models [5–12] and demonstrated experimentally in large crocodiles [10,13]. The argument that homeothermy can be attained at low energy cost in large dinosaur through gigantothermy is compelling. These ideas appear in high-impact literature [14–19]. The implication is that, if an ectotherm can achieve a high body temperature, then it does not need to be an endotherm.

Estuarine crocodiles ( *Crocodylusporosus* ) have been specifically used as models for large, ectothermic dinosaurs [10,11]. Body temperatures of large (up to 1 tonne) crocodiles can average above 30°C in tropical Queensland. Based on this, Seebacher et al. [10] estimated that a 10 tonne dinosaur could have a stable body temperature above 31°C without endothermy in a similar climate, even in winter. They proposed that natural selection for high metabolic rates of endothermy would be diminished if high body temperature could be attained without the energy cost typical of endotherms. It is clear that one advantage of a high and stable body temperature is coordination of biochemical and physiological activities at optimum levels, an explanation often used in relation to endotherms. Seebacher et al. also recognised that warm, ectothermic reptiles nevertheless do not show the same capacity for sustained activity levels characteristic of endotherms, but the difference in performance would be smaller if they were warmer. This is undoubtedly true, but it would be interesting to know how much smaller it would be.

Moreover, it would be more interesting to determine the total power output, including both aerobic and anaerobic sources, to assess how ectothermic, crocodile-like dinosaurs would compare to endothermic, mammal-like dinosaurs. *Aerobic metabolic scope* is the energy production by the respiratory metabolic pathways, as measured by the difference between resting and maximum rates of O_2_ consumption. *Anaerobic metabolic scope* is the maximum rate of useful energy production by anaerobic glycolysis, as measured by the rate of lactate production. Both measurements can be converted to ATP production and then into power, measured as a rate in units of Watts (Joules per second). Anaerobic scope, which is a rate, should not be confused with *anaerobic capacity*, which is the total amount of energy produced anaerobically by the time of total exhaustion [20].

The literature on anaerobic scope in vertebrates is not particularly rich, because of the practical difficulties in measuring the rate of anaerobic energy production. Whereas aerobic energy can be determined easily by measuring O_2_ consumption rate of exercising animals, anaerobic energy must be measured by rates of lactate production in muscle as determined by muscle biopsy, whole body homogenization or, more indirectly, blood lactate levels before and after exercise. Data from small reptiles and rodents indicate that maximum total power outputs during 5 min of burst activity are similar [21]. Ectotherms can be as powerful as endotherms during sprint locomotion [22,23], but ectotherms do it largely anaerobically, with white muscle and few mitochondria. Reptiles can be 95% anaerobic during strenuous activity, and anaerobic metabolic scope can be 2-5 times higher than the aerobic scope [24]. This is impressive, because the anaerobic pathway produces only about 10% of the ATP energy as the aerobic one from the same amount of substrate. However, all of these conclusions come from small reptiles and mammals and may not represent the situation in crocodiles or dinosaurs that weighed 3–5 orders of magnitude more. This paper addresses this question in the estuarine crocodiles, *Crocodylusporosus* , weighing up to 200 kg, because this is approximately the mass range available from an earlier study of anaerobic metabolism of this species [25]. It shows that total power generation in maximally active crocodiles is low compared to mammals of the same size and that the disparity increases greatly in larger animals.

## Methods and Results

In this presentation metabolic power is the rate of energy production by the whole animal and is measured in Watts. Muscular power may be calculated on the basis of the energy content change of 30.5 kJ mol^-1^ when adenosine triphosphate (ATP) is converted to adenosine diphosphate (ADP) during muscle contraction [26]. ATP is regenerated from three sources: (a) aerobic respiration, (b) anaerobic glycolysis and (c) phosphocreatine (PCr) anaerobically in the muscle.

### Aerobic energy production in crocodiles

Aerobic power is derived from oxidation of substrates through aerobic biochemical pathways in the cytoplasm and mitochondria (glycolysis, tricarboxylic acid cycle, electron transport chain) in all cells that have sufficient O _2_ available. Ultimately, the energy in the substrates leaves the body as external work and heat. In animals in steady state that are not performing external work, the energy output can be measured directly as heat or indirectly as O _2_ consumption rate. However, the caloric equivalent of O _2_ consumption of 21.1 kJ L^-1^ cannot be used to measure power available to the muscles, because conversion to ATP is not 100% efficient. Therefore, assuming that 30 mol of ATP (rather than the old, theoretical value of 36) is produced from the oxidation of 1 mol of glucose moiety and 6 mol of O_2_ [27], 0.22 mol ATP L O_2_^-1^ results. At 30.5 kJ mol^-1^, this represents 6.81 kJ L O_2_^-1^. Bennett and Ruben [21] used a higher value of 0.29 mol ATP L O_2_^-1^, or 8.85 kJ L O_2_^-1^, possibly because energy conversion was thought to be more efficient.

Standard metabolic rate (SMR, ml O_2_ min^-1^) was measured at 30°C in 44 captive crocodiles in relation to body mass (M, kg) [28]. The measurements were made under carefully controlled conditions, over several days in post-absorptive animals. Within a mass range of 3.3 orders of magnitude (0.19–389 kg), the allometric relationship was: SMR = 1.01M^0.829^. The 95% confidence interval of the slope of the log-transformed version of this equation was 0.803–0.855. The power (P, Watts) equation becomes: P = 0.115 M^0.829^.

Aerobic maximum metabolic rate (MMR) is calculated assuming that MMR is 6.56 ml O_2_ min^-1^ in a 1 kg animal, which is the mean of two studies of *C* *. porosus* [29,30] and four studies of *A* *. mississippiensis* [31–34] that all involved running locomotion. MMR is assumed to scale with body mass to the same exponent (0.829) as SMR. This exponent is similar to the mean of 0.839 measured for MMR in small (0.049–4.078 kg) *C* *. porosus* at different temperatures [29]. Unfortunately, measurements from larger crocodiles are not available for practical reasons. Therefore the best available equation is: MMR = 6.56M^0.829^. The power equation becomes: P = 0.744 M^0.829^.

### Anaerobic energy production in crocodiles

At higher levels of activity, the aerobic pathway cannot keep up with energy demand, so some ATP is generated anaerobically in glycolysis, drawn mainly from glycogen stores and leading to lactate in the muscle. 1 mol of glucose moiety from glycogen generates 3 mol of ATP and 2 mol of lactate. Thus 45.75 kJ mol lactate^-1^ is produced. Bennett and Ruben [21] used 45.9 kJ mol lactate^-1^.

**Figure 1:**
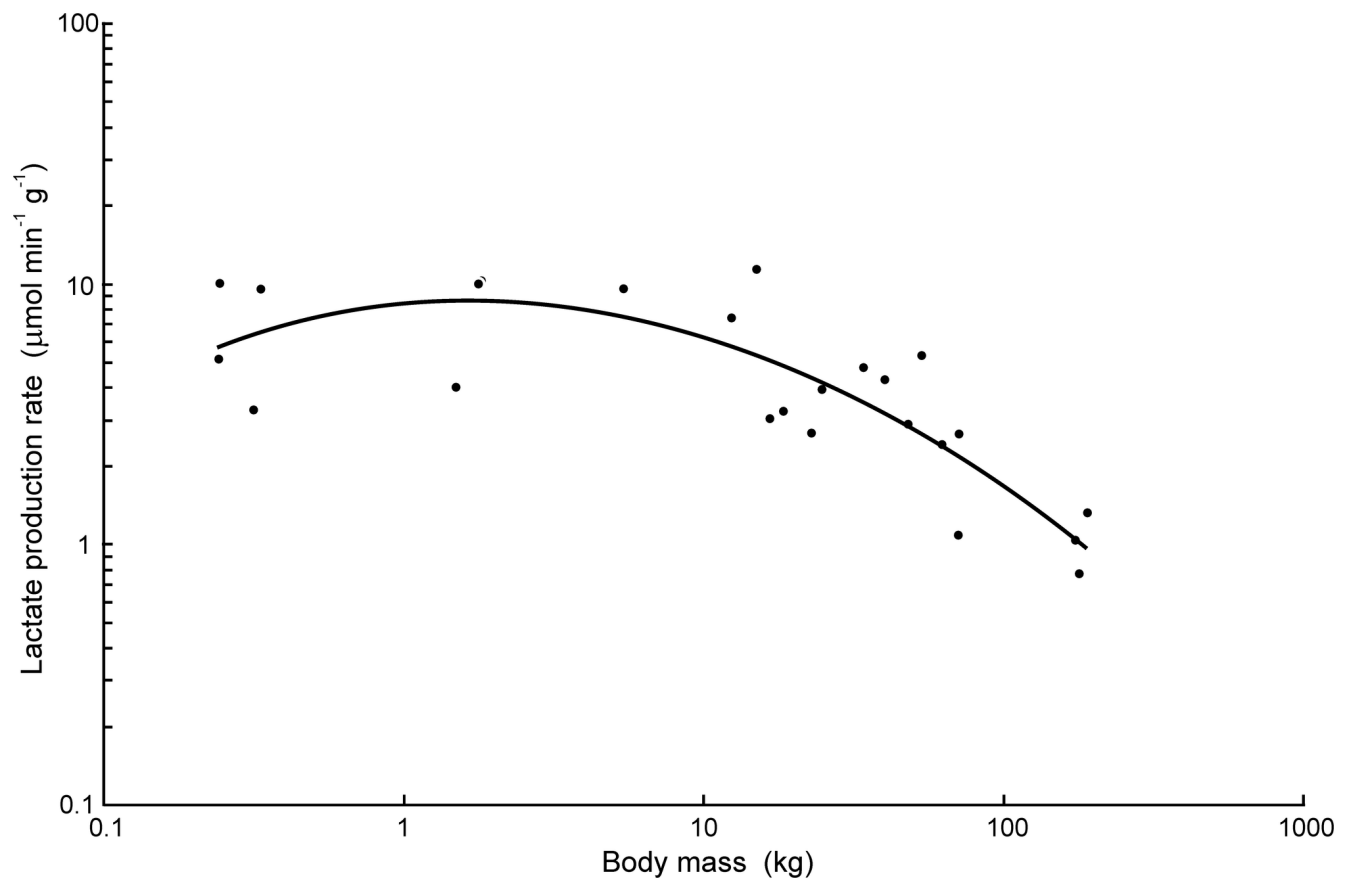
Mean rate of lactate production in exercising *Crocodylusporosus* . Data are given as rates per gram of muscle and in relation to body size in 24 animals. Data from [25]. A 3-parameter regression is set to the data (see text).

Early research on small reptiles involved whole body homogenization in a blender to analyse lactate accumulation at the end of exercise, e.g. painted turtles [35]. This approach is clearly impossible for large animals. Anaerobic energy production in *C* *. porosus* at 30°C is therefore calculated from the rate of lactate appearance (μmol min^-1^) in the muscle and blood during exhaustive exercise, as measured in a previous study [25]. Both blood and muscle are required, because lactate levels are not equal in them. Wild crocodiles (ranging in mass from 0.24–188 kg) were approached by boat at night and secured to a thin cord by a barb through the skin. This caused the animals to thrash violently to the point of complete exhaustion in water, and they failed to right themselves on landing. The duration of exercise was measured and blood and tail muscle samples were obtained at exhaustion. Lactate concentrations [L] in the muscle and blood were different, so were used to measure anaerobic energy production, assuming that the muscles occupied 50% of the body mass [36]. The rate of lactate accumulation per gram of muscle is shown in Figure 1. The rate was independent of body size in smaller animals, but decreased in larger ones. A 3-parameter equation set to the data is: log d[L]/dt = -0.2227 logM^2^ + 0.1048 logM + 0.9107 (R^2^ = 0.70). The rate of lactate production was reflected in the rate of glycogen depletion in muscles that decreased in larger animals and averaged only about 15% lower than total lactate production. Also consistent was a decreasing activity of muscle phosphorylase (the enzyme responsible for the conversion of muscle glycogen to glucose-6-phosphate that begins glycolysis anaerobically) in larger animals.

**Figure 2:**
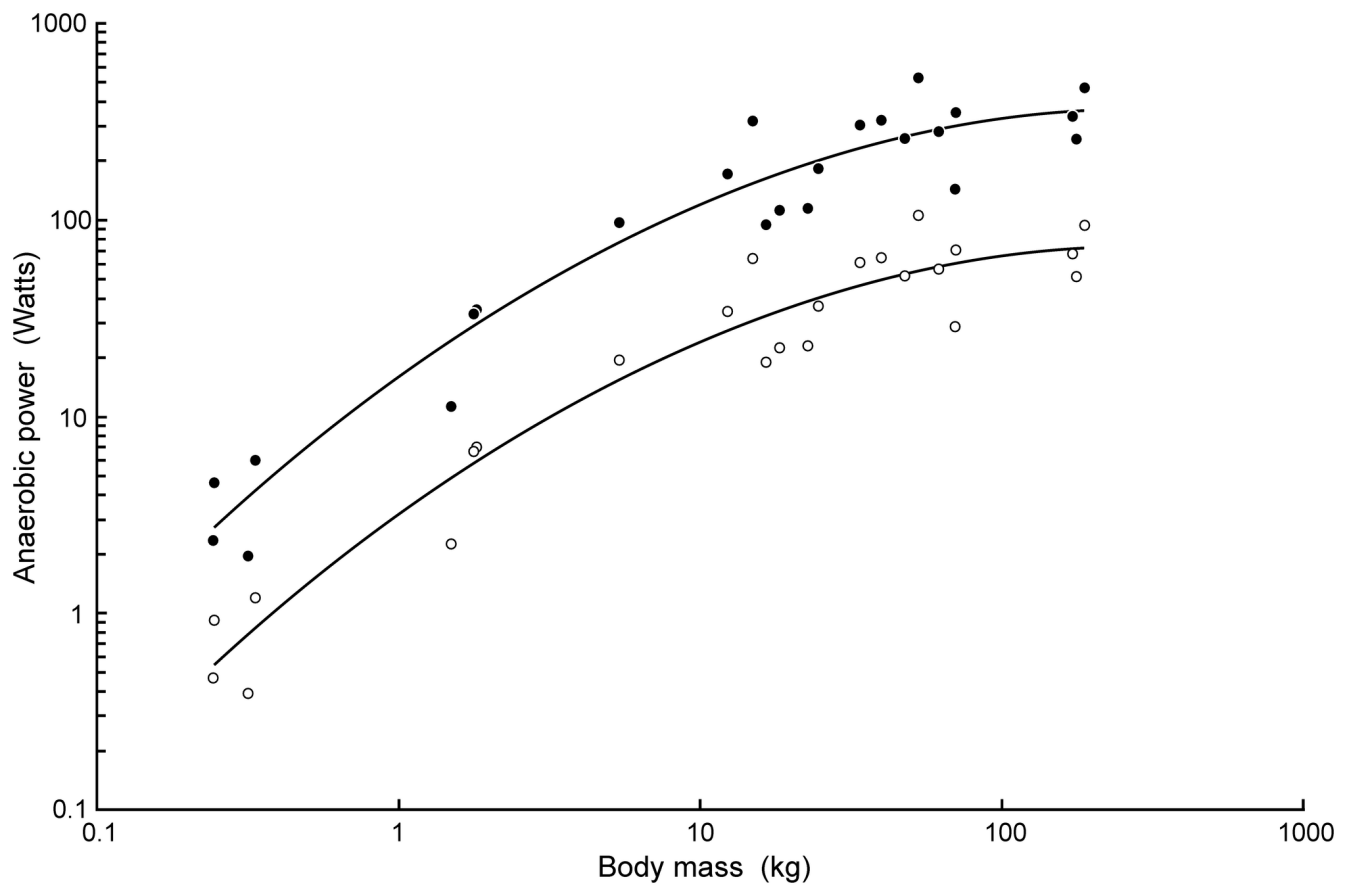
Rate of anaerobic power generation in *Crocodylusporosus* in relation to body mass. Lower data set (open circles) is the measured mean rate over the entire course of exercise to fatigue [25]. Upper data set (filled circles) is the calculated burst rate during the first 10% of the exercise period, assuming that the rate decreases exponentially to zero at exhaustion. This multiplies the mean rate by a factor of 5. The curves are 3-parameter regressions set to the data (see text).

**Figure 3:**
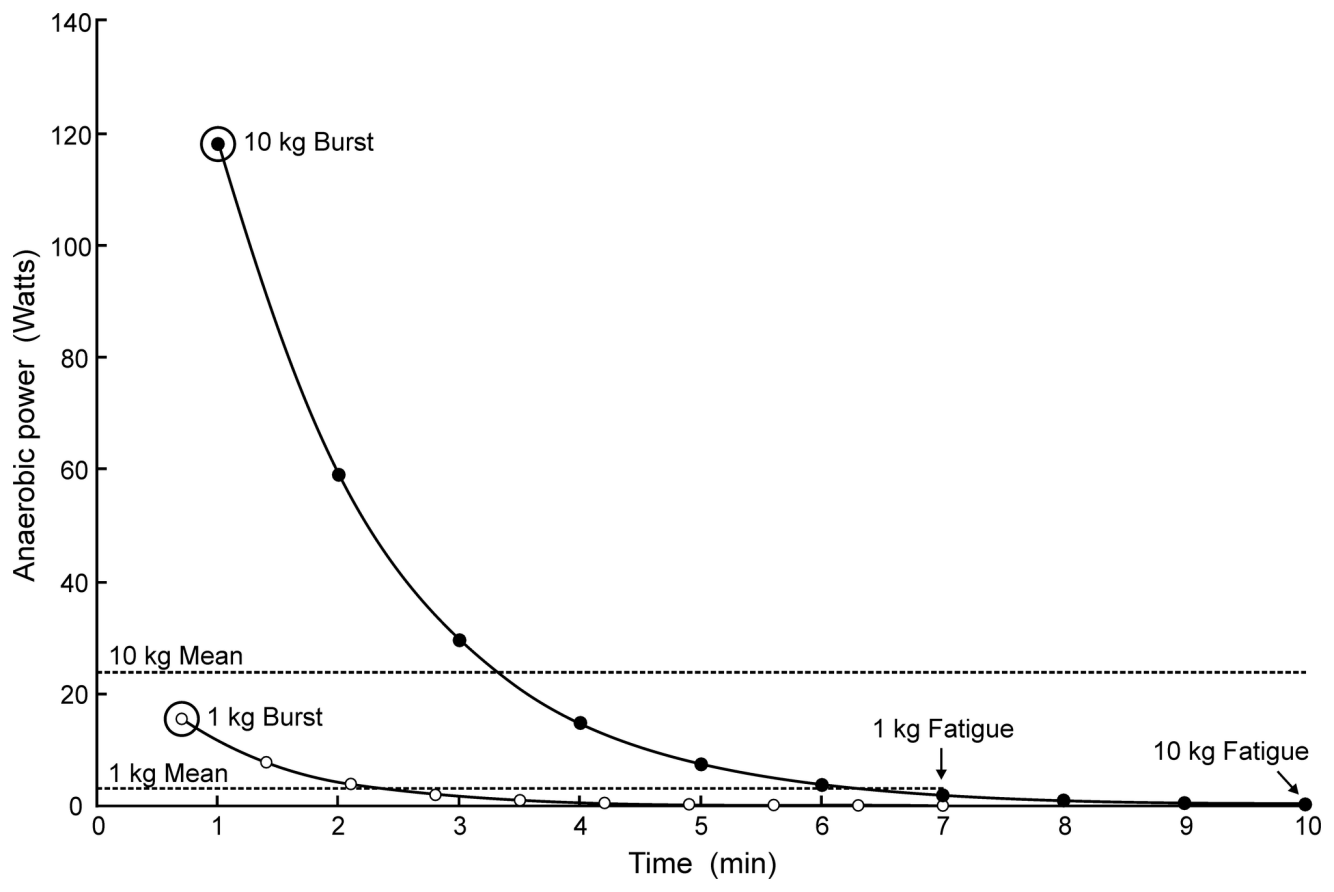
Rate of energy production (power) from anaerobic glycolysis during exhaustive exercise in *Crocodylusporosus* . To fit on the figure, only 1 kg and 10 kg body masses are plotted (data not shown for larger animals). The total energy produced anaerobically during the entire exercise period is related to the area under each curve. Horizontal lines indicate mean anaerobic power to the point of fatigue. Curves are assumed exponential decreases in power during the exercise period. The highest points on the left represent burst power during the first 10% of exercise and are used to estimate the maximum initial anaerobic contribution to exercise. Data are derived from [25].

According to these measurements, the rate of power generation from anaerobic glycolysis increases with body size non-linearly on log-transformed axes (Figure 2). The equation for mean power is: log P = -0.2227 logM^2^ +1.1048 logM +0.4919(R^2^ = 0.94). However, it is unreasonable to expect that the rate of anaerobic metabolism is constant throughout exercise until the point of exhaustion. It is more reasonable to consider an exponential decrease in the anaerobic component, because the exercising crocodiles lost intensity as they exercised. The rate of glycolysis decreases during exercise in juvenile American alligators, *Alligator* *mississippiensis* [37,38], and sprint performance in humans decreases progressively during longer runs [39,40]. We know that *C* *. porosus* become completely fatigued following approximately 7, 10, 30 and 50 min in animals weighing 1, 10, 100 and 200 kg, respectively [41]. If each exercise period is divided into 10 equal intervals and the rate of energy production calculated during each interval adds up to the total anaerobic energy produced during the entire period, we can estimate burst anaerobic energy production. If one accepts the first 10% of the exercise period as the maximum and assumes an exponential decrease over the exercise time, the maximum turns out to be 5 times higher than the mean (Figure 3). The equation for burst power is: log P = -0.2227 logM^2^ +1.1048 logM +1.1909(R^2^ = 0.94).

### Aerobic and anaerobic energy production in mammals

Standard (basal) metabolic rate (ml O_2_ min^-1^) of mammals scales with body mass (kg) according to the equation: SMR = 8.85M^0.676^ [42]. Converted to power: P = 1.00M^0.676^.

Maximum aerobic metabolic rate (ml O_2_ min^-1^) of placental mammals scales with body mass (kg) according to the equation: MMR = 118.2M^0.872^ [43]. Converted to power: P = 13.41M^0.872^.

Maximum anaerobic metabolic rate has not been analysed allometrically in mammals, but it is said that maximum running speeds of mammals average 2.12 times faster than their maximum aerobic speeds [23]. Knowing that energy expenditure is fairly linearly related to speed in mammals, the energy cost of aerobic running can be extrapolated past the maximum aerobic speed to the maximum speed. Thus the anaerobic component of maximum speed can be assumed to be 1.12 times the aerobic power production rate, and the power equation becomes: P = 15.02M^0.872^.

### Comparison of crocodiles and mammals

**Figure 4:**
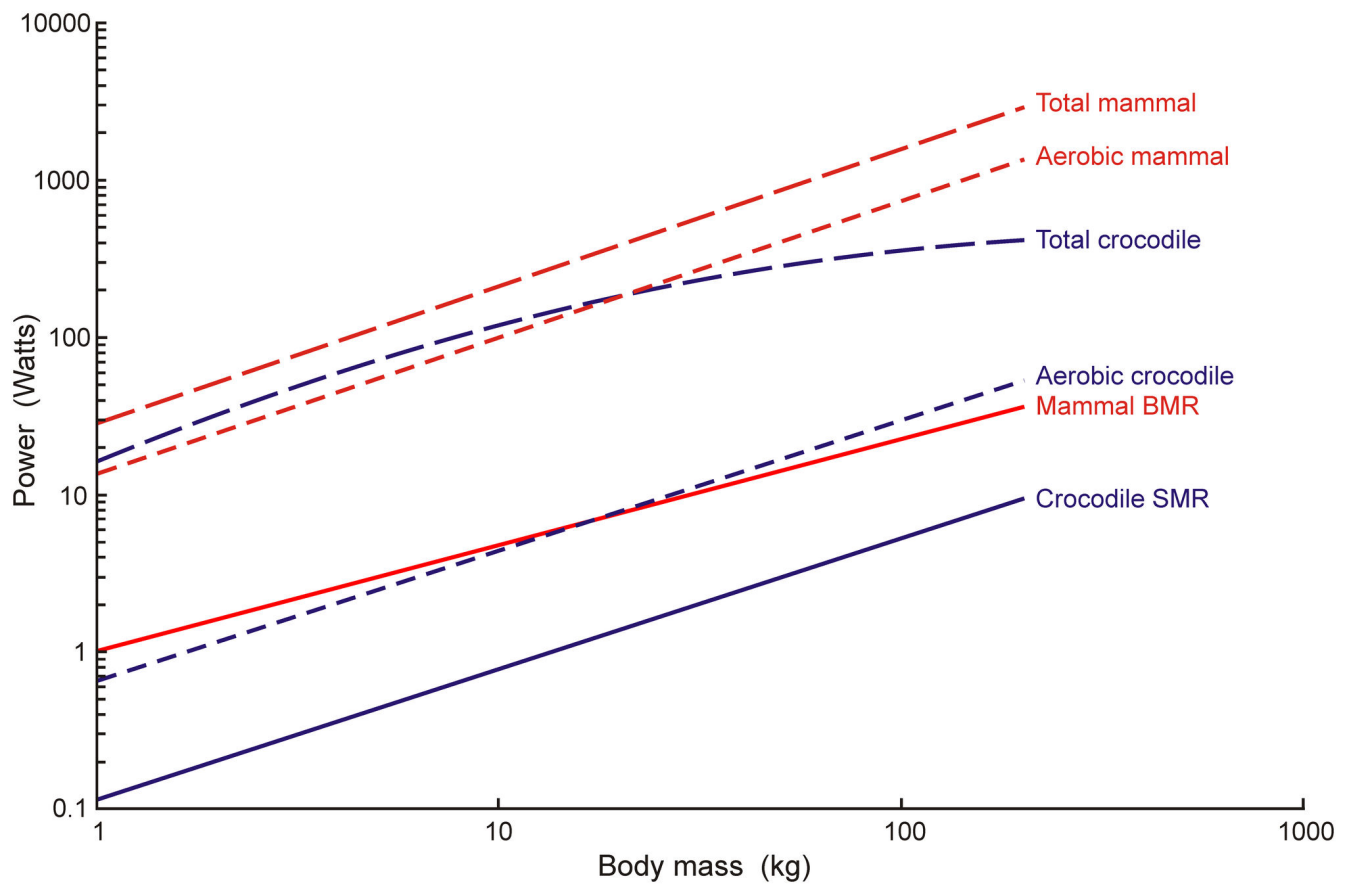
Allometric analysis of power output in *Crocodylusporosus* compared to mammals of the same size. *SMR* is the standard metabolic rate, *Aerobic* is the aerobic power and *Total* is the sum of aerobic and anaerobic power output. Equations for the lines are provided in the text.

**Figure 5:**
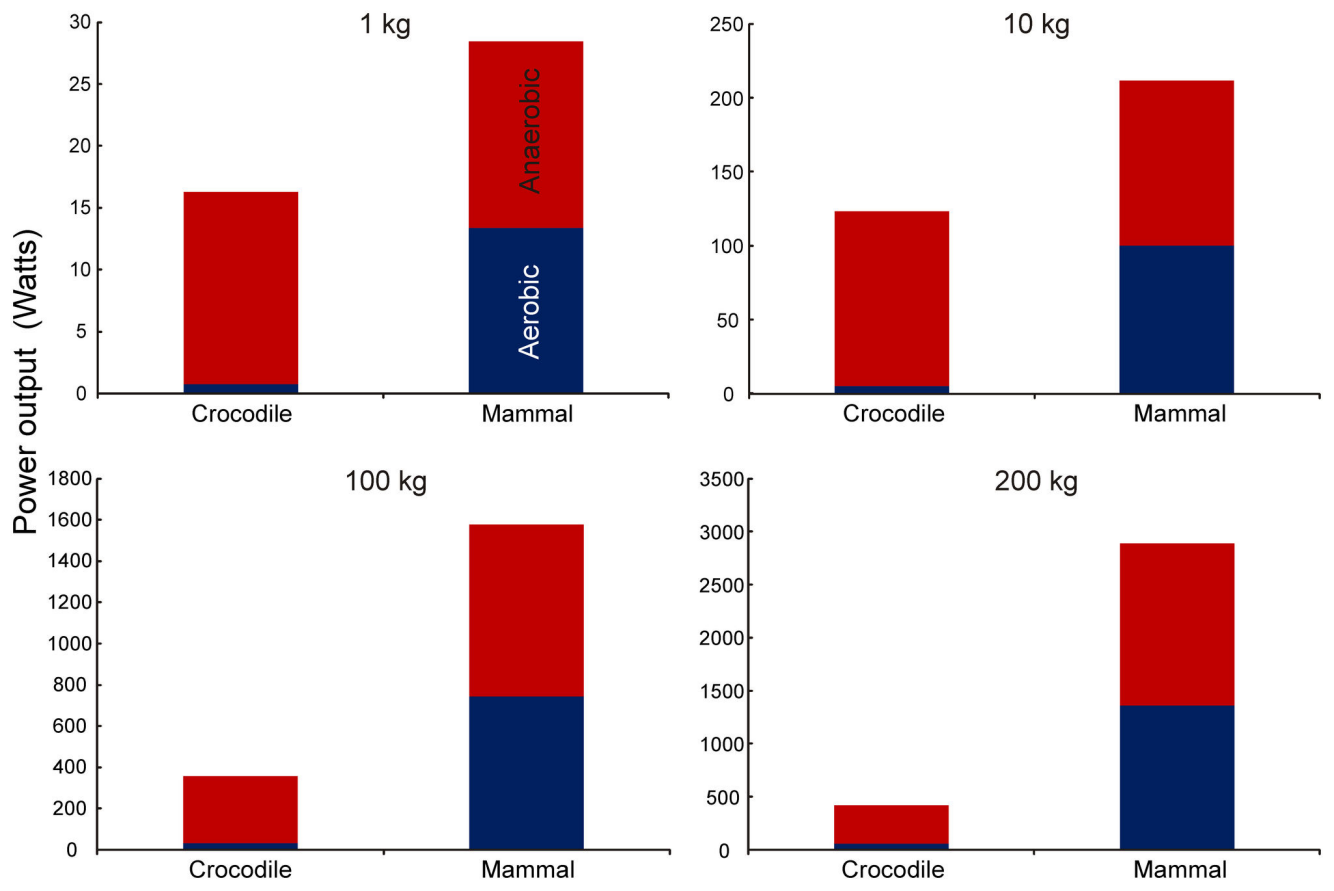
Total power output in *Crocodylusporosus* compared to a mammal of the same size. Aerobic (blue bottom) and anaerobic (red top) fractions of the total are given for animals weighing 1, 10, 100 and 200 kg.

The equations for aerobic and anaerobic power are added to arrive at a total power (Figure 4). On arithmetic axes, the differences between crocodiles and mammals are clearer (Figure 5).

Some energy is held as phosphocreatine (PCr), but it is not included in the analysis. There are two reasons for this exclusion. First, the concentrations of PCr seem to be similar in reptiles and mammals. PCr levels average 22.7 mM in red, and 37.3 mM in white, iliofibularis muscle *in vivo* in resting lizards, *Dipsosaurus* *dorsalis* [44]. Resting muscle of American alligators was assumed to contain 27 mM PCr [45]. By comparison, human muscle contains 16–32 mM [46] and racehorses reach about 65 mM [47]. Secondly, PCr energy is exhausted quickly, typically during the first seconds of burst activity, and produces much less energy relative to aerobic and anaerobic energy sources [48]. It is quite possible that PCr can produce equivalent power in crocodiles and mammals during the first seconds of activity, consistent with observations on similar sprint speeds of lizards and mammals [22,23], but after about a minute, PCr ceases to be a source of energy.

## Discussion

Estuarine crocodiles appear to be extremely powerful animals. They increase their exercise endurance and tolerances to high lactate and acidosis as they get larger [41], particularly after they reach adulthood, when they engage in territorial and courtship fighting [49]. The crocodiles weighing 100–200 kg violently resisted capture for up to 48 min in our earlier study, and larger animals can struggle for 1–2 hours (GJW Webb, pers. comm.). They produce the highest level of blood lactate known for any animal as a result of activity to fatigue [41].

Although the anaerobic capacity (the total anaerobic energy produced) of crocodiles is high, the anaerobic scope (the rate of anaerobic energy production) is not particularly high. According to this analysis, a 1 kg crocodile has about the same anaerobic scope as a 1 kg mammal, but the scope decreases with increasing body size (Figure 5). Thus, total energy production in a 1 kg crocodile is 57% of that of a mammal, decreasing to 14% at 200 kg (Figure 5). These estimates align with the earlier conclusion that total aerobic and anaerobic performance in reptiles and rodents were similar, because those animals were small, weighing less than 262 g [21]. If the trend continues in crocodiles larger than 200 kg, then the disparity may increase further.

### Critique of assumptions

It might be argued that the aerobic contribution to power is underestimated in larger crocodiles. The present analysis is based on small ones, and the assumption of the same scaling exponent as SMR is wrong. It is known that MMR in mammals and birds scales with a higher exponent than SMR [50]. However, increasing the exponent has a relatively small effect here. With the current equation, a 200 kg crocodile is expected to produce 60 Watts by aerobic metabolism. If the exponent is arbitrarily raised to 1.0, then the aerobic power becomes 149 Watts and the total power 507 Watts. In comparison, a 200 kg mammal produces 1524 Watts by aerobic metabolism, and a total of 2886 Watts. Even if one assumes zero anaerobic metabolic scope for the mammal, the 200 kg crocodile is only one-third as powerful. But the anaerobic scope of mammals is assumed to be 1.12 times the aerobic scope, according to Garland [23]. However, the anaerobic scope of mammals is probably closer to 2.37 times the aerobic scope. This value comes from the ratio of maximum speed estimated from a 3-parameter regression of Garland [23] to maximum aerobic speed of 12 species of mammals weighing between 0.583–254 kg [51]. The mean ratio is 3.37 and the range is 2.13–4.67.

Similarly, the values for aerobic MMR in crocodilians might be argued to be underestimated, as in most cases, the studies involved running rather than swimming, and they did not specifically demonstrate that MMR was reached. However, we know that *C* *. porosus* was exercised to fatigue in Wright’s study [29], which indicates that the cardiorespiratory system was operating at its maximum capacity. This would set the limit on any aerobic activity, including swimming. Moreover, because the aerobic power is less than 13% of total power in crocodiles (Figure 5), even a large error would have a small effect on total power.

It might be noticed that the crocodile body temperature is assumed to be 30°C while the mammal is 38°C. If one assumes a Q_10_ of 2.6 for *C* *. porosus* [29], then a crocodile operating at 38°C would have an aerobic power of 129 Watts and an anaerobic power of 769 Watts, giving a total of 898 Watts, which is still less than one-third of the total for a mammal. However, large *C* *. porosus* do not achieve 38°C body temperatures in nature, so the comparison is moot.

The comparison of crocodiles with mammals may appear unfair, because crocodiles are usually considered rather slow cruisers in water and usually rather sedentary on land, whereas the mammals for which maximal metabolic rate is available might be considered animal athletes (e.g., dogs, goats, cows, horses). If the mammals had been restricted to sluggish ones (e.g., echidnas, pangolins, sloths, elephants), their aerobic metabolic scope would probably have been lower. Data for aerobic and anaerobic scope for these animals are unfortunately not available, but neither are data from sluggish reptiles (e.g., tortoises, shingleback lizards, gila monsters). It is noteworthy that sluggish mammals and reptiles necessarily use passive defences (e.g., armour, spines, venom) rather than active defence (e.g., running, fighting). Although crocodiles are to some extent armoured, adult crocodiles actively defend territories by vigorous fighting, sometimes to death [52]. A high capacity for anaerobic power generation is extremely important for survival.

### Relevance to dinosaurs

It is clear that using a large, warm, inertially homeothermic crocodile as a model for dinosaurs produces a relatively weak animal that also has very poor endurance compared to a good endotherm. Total power production of small crocodiles is less than in mammals, mainly because of a low absolute aerobic metabolic scope (Figure 5). Adding anaerobic energy in larger crocodiles does not even get total power up to the level that mammals can generate using aerobic pathways alone. Thus a large mammal could sustainably produce more power than a crocodile exercising unsustainably. It is tempting to imagine a fight between a crocodile-like dinosaur and a mammal-like dinosaur on otherwise equal terms. It is clear which would have the advantage. This may explain why there are no reptiles that act like cats and chase down mammalian prey. If dinosaurs had similar exercise physiology as modern crocodiles, then it is unlikely that they would have been as successful as predators and prey for 185 million years, while coexisting with mammals in completely terrestrial environments.

This analysis upholds Bennett and Ruben’s aerobic capacity model for the evolution of endothermy [21]. The main idea behind this theory is that endothermy evolved in parallel with sustainable aerobic activity rather than simply as a means to raise the body temperature. It is clear from this study that high body temperature is not enough to develop the power and endurance of endothermy. What is necessary is enhancement of aerobic capacity by investing the muscles with mitochondria. Total mitochondrial surface area in mammals is four times higher than in reptiles [53], and sustaining such metabolic machinery requires higher maintenance costs, apparent in high standard metabolic rate. Mitochondria constantly lose energy by leaking protons across the inner membrane at a rate of about 20% of the resting metabolic rate in mammals [54]. They produce even more energy at rest to maintain Na^+^/K^+^ gradients across membranes [55]. Thus maintenance energy and inherently leaky membranes ultimately produce heat energy that could be either wasted in an ectotherm or useful in an endotherm to raise body temperature and enable physiological thermoregulation.

Interestingly, the archosaurs split into two lineages in the Middle Triassic. The Crurotarsi (crocodilian line) diversified greatly by the Jurassic, when there were hundreds of genera in wholly terrestrial and aquatic (marine and freshwater) habitats. These crocodilians were probably endotherms and competed with endothermic dinosaurs. There is much physiological, anatomical and developmental evidence that the ancestors of all crocodiles were highly aerobic and endothermic predators. Modern crocodiles have 4-chambered hearts and flow-through lungs that are usually found in endotherms, but have a low gas transport capacity characteristic of ectotherms [3,56]. Aside from the heart and lungs, they have many other features indicating an endothermic ancestry, including lung ventilation during locomotion [57], fibrolamellar bone in neonates and juveniles [58,59] a fast evolutionary molecular clock [60] and the ability for galloping locomotion [61]. Evidence from embryonic heart development indicates that sometime in the crocodilian lineage, one group apparently lost the completely divided pulmonary and systemic circuits of endotherms and developed *de novo* the ability bypass the lungs, a characteristic of diving reptiles that can extend dive duration [56]. Living crocodiles are archetypal, sit-and-wait predators in water that grab their unsuspecting prey with a short burst of power and immediately crush and eat small ones or drown large ones before eating. They have no need for endothermy or sustained locomotion. They have small nutrient foramina on the femoral shaft, indicative of low levels of activity, in contrast to mammals, birds and dinosaurs [62]. Thus the sit-and-wait aquatic predator niche of modern crocodilians has selected for a shift from endothermy to ectothermy, and from mainly aerobic to anaerobic power, with a consequent reduction in maximal power output to a fraction of that expected for aerobic endotherms.
